# Vitamin D supplementation and mortality risk in chronic kidney disease: a meta-analysis of 20 observational studies

**DOI:** 10.1186/1471-2369-14-199

**Published:** 2013-09-25

**Authors:** Zhenfeng Zheng, Huilan Shi, Junya Jia, Dong Li, Shan Lin

**Affiliations:** 1 Nephrology Department, General Hospital of Tianjin Medical University, No.I54 Anshan Road, Heping District, Tianjin 300052, China; 2 Radiology Department, General Hospital of Tianjin Medical University, No.I54 Anshan Road, Heping District, Tianjin 300052, China

**Keywords:** Vitamin D, Mortality, Calcitriol, Paricalcitol, Chronic kidney disease

## Abstract

**Background:**

Vitamin D insufficiency correlates with mortality risk among patients with chronic kidney disease (CKD). The survival benefits of active vitamin D treatment have been assessed in patients with CKD not requiring dialysis and in patients with end stage renal disease (ESRD) requiring dialysis.

**Methods:**

MEDLINE, Embase, the Cochrance Library, and article reference lists were searched for relevant observational trials. The quality of the studies was evaluated using the Newcastle-Ottawa Scale (NOS) checklist. Pooled effects were calculated as hazard ratios (HR) using random-effects models.

**Results:**

Twenty studies (11 prospective cohorts, 6 historical cohorts and 3 retrospective cohorts) were included in the meta-analysis., Participants receiving vitamin D had lower mortality compared to those with no treatment (adjusted case mixed baseline model: HR, 0.74; 95% confidence interval [95% CI], 0.67-0.82; *P* <0.001; time-dependent Cox model: HR, 0.71; 95% CI, 0.57-0.89; *P* <0.001). Participants that received calcitriol (HR, 0.63; 95% CI, 0.50-0.79; *P* <0.001) and paricalcitol (HR, 0.43 95% CI, 0.29-0.63; *P* <0.001) had a lower cardiovascular mortality. Patients receiving paricalcitol had a survival advantage over those that received calcitriol (HR, 0.95; 95% CI, 0.91-0.99; *P* <0.001).

**Conclusions:**

Vitamin D treatment was associated with decreased risk of all-cause and cardiovascular mortality in patients with CKD not requiring dialysis and patients with end stage renal disease (ESRD) requiring dialysis. There was a slight difference in survival depending on the type of vitamin D analogue. Well-designed randomized controlled trials are necessary to assess the survival benefits of vitamin D.

## Background

Mineral and bone disorders (MBD) are early and common complications of CKD, and progress as glomerular filtration rate (GFR) declines. Multiple factors contribute to the development and maintenance of CKD-MBD, but principally involve phosphate retention and vitamin D metabolism abnormalities. *Kidney Disease: Improving Global Outcomes* defines chronic kidney disease-mineral and bone disorder (CKD-MBD) as a systemic syndrome characterized by abnormalities in serum calcium, phosphorus and parathyroid hormone (PTH) concentration, vitamin D metabolism, and bone turnover [1]. This syndrome is common among CKD patients and has been associated with an increased risk of cardiovascular calcification [2,3] and mortality [4]. The Third National Health and Nutrition Examination Survey (NHANES III) reported 15068 adults patients with vitamin D deficiency and demonstrated a higher prevalence of cardiovascular disease and mortality in untreated patients [5]. An association between vitamin D deficiency and other traditional cardiovascular risk factors, such as hypertension, insulin resistance, diabetes, and dyslipidemia, has also been reported [6,7]. The recognition of biochemical components of CKD-MBD associated with increased mortality in dialysis patients [8] and in patients with CKD not treated with dialysis [9] has provided an impetus to explore the effect of these factors on survival and associated treatment modalities. Numerous reports have characterized the nonskeletal benefits of vitamin D [10].

Wang et al. and Pittas et al. reported the benefits of vitamin D supplementation on cardiovascular disease (CVD) in the general population [11,12]. Nutritional vitamin D supplementation has also been reported to be beneficial to CKD patients [13]. Most reviews, however, had few participants, short follow-up, and lacked survival analyses. We conducted a systematic review of the literature to assess whether vitamin D supplements reduced mortality in patients with ESRD on dialysis and patients with CKD not requiring dialysis.

## Methods

### Data sources and Search strategy

MEDLINE (1966 to March 2013), EMBASE (1980 to March 2013) and the Cochrance Controlled Trials Register (CCTR-Specialized Renal Registry) were searched. Relevant studies were identified [14,15]. References from identified studies were reviewed to find additional relevant studies. This systematic review was planned, conducted, and reported following the Meta-analysis of Observational Studies in Epidemiology (MOOSE) guidelines [16].

### Eligibility criteria

Studies were included in the meta-analysis if they met the following criteria: (1) cohort study design and follow-up duration was at least 1 year; (2) patients had chronic kidney disease or renal replacement treatment; (3) patients were treated with active vitamin D sterols (alfacalcidol, doxercalciferol, calcitriol, maxacalcitol, falecalcitriol and paricalcitol), but not native vitamin D (ergocalciferol and cholecalciferol); (4) the outcome of interest was all-cause mortality or cardiovascular mortality; (5) there was quantitative data (i.e., events rates, risk ratio [RR] or hazard ratio [HR]). If data were duplicated in more than 1 study, we included the study with the largest number of patients.

### Data extraction

All data were independently abstracted by 2 investigators (Z.F.Z. and H.L.S) using a standardized data collection form. Discrepancies were resolved through discussion with other investigators (D.L. and J.Y.J.) and through reference to the original articles.

### Quality assessment

Two authors (Z.F.Z. and H.L.S.) independently evaluated the quality of each study using the 9-star Newcastle-Ottawa Scale (NOS) [17]. The Strengthening the Reporting of Observational Studies in Epidemiology (STROBE) checklist for cohort studies was used to limit heterogeneity resulting from differences in study design [18]. Disagreements were resolved by consensus.

### Statistical analysis

Studies that provided relative risk (RR) or hazard ratios (HR) were used directly in the pooled meta-analysis calculations. Overall crude (unadjusted) HR and adjusted HR were calculated. Adjusted variables included demographic and clinical values, biochemical indices and erythropoietin and phosphate binder use. The overall pooled-effect estimates were calculated using DerSimonian & Laird random-effect models. The Q test was used to assess the presence of heterogeneity and the I^2^ index was used to quantify the extent of heterogeneity [19,20]. I^2^ values of 25% or less indicated low heterogeneity, values near 50% indicated moderate heterogeneity, and values 75% or greater indicated high heterogeneity [21]. Publication bias was assessed using funnel plots for each outcome and ln(HR) was plotted against its standard error. The Begg rank correlation test was used to examine asymmetry of the funnel plot [22]. The Egger weighted linear regression test was used to examine the association between mean effect estimate and its variance [23]. If an asymmetric funnel plot was found, a contour-enhanced funnel plot was used to further explore the source of bias [24]. P<0.05 was considered statistically significant. All tests were 2-sided. All analyses were conducted using STATA version 12.0 (StataCorp, College Station, Texas).

## Results

### Literature search

**Figure 1:**
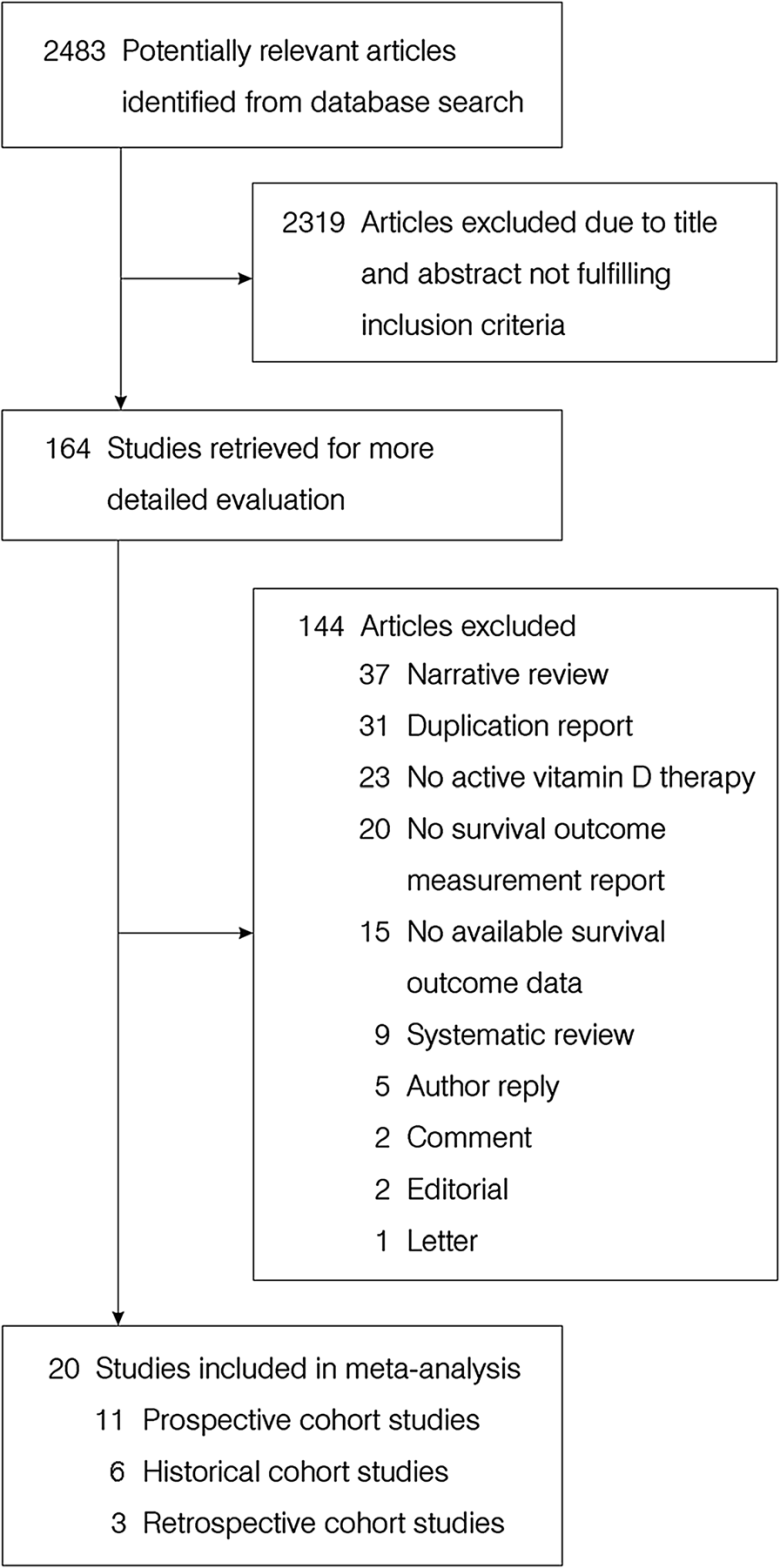
Selection process for studies included in the meta-analysis.

Our initial literature search yielded 2483 citations. 2319 articles were excluded. The majority of these citations were excluded at the level of title or abstract review. There were 164 citations which were considered to be potentially eligible. 144 articles were excluded after reviewing the article. Excluded articles included 37 narrative reviews, 31 duplication studies, 23 without vitamin D treatment, 20 without survival outcome, 15 without survival outcome data, 9 systematic reviews or meta-analyses, 5 author replies, 2 comments, 2 editorials and 1 letter. Twenty studies were considered eligible to be included in the meta-analysis [25-44]. The overall search flow is presented in Figure 1.

### Study characteristics

The characteristics of eligible studies are summarized in Table 1. Of the 20 included observational studies, eleven were prospective cohort studies. [26,28,31,34-36,39,41-44]. The remaining 9 consisted of 6 historical cohort studies [25,27,29,30,32,33] and 3 retrospective cohort studies [37,38,40]. Seventeen studies reported ESRD patients on dialysis [25-30,32,33,35-39,41-44] and three reported CKD patients not on dialysis [31,34,40]. Five studies compared calcitriol to no active vitamin D treatment [28,31,34,38,42], two studies compared paricalcitol to no active vitamin D treatment [33,42] and four studies compared alfacalcidol to no active vitamin D treatment [26,40,41,44]. Nine studies did not report the specific analogues used and compared active vitamin D compounds with no treatment [27,29,30,32,35-37,39,43]. Two studies compared the survival benefits of paricalcitol and calcitriol [25,30]. Several sophisticated statistical models were used in these observational studies. Fifteen studies used a fixed covariate baseline Cox model [25,26,30-35,37-41,43,44], two studies used a time-dependent Cox model [27,42], and three studies used both Cox models [28,29,36]. Only 4 studies were confirmed by intention to treat (ITT) analysis [27,30,32,34].

**Table 1:** Observational studies examining active vitamin D administration in patients with CKD or on dialysis

**Author**	**Year**	**Country**	**# Participants**	**Study period**	**Patient category**	**Treatment**	**Comparator**	**Vitamin D dosage**	**Study design**	**Statistical methods**	**Follow-up duration months**	**ITT analysis**	**NOS scale**
Teng et al.	2003	United States	67399	1999 to 2001	Prevalent HD patients	Paricalcitol	Calcitriol	NA	Historical cohort multicenter study	Baseline Cox model; as-treated analysis	36	no	6
Shoji et al.	2004	Japan	242	1992 to 1998	Prevalent HD patients	Alfacalcidol	No treatment	NA	Prospective cohort single center study	Baseline Cox model	76	no	9
Teng et al.	2005	United States	51037	1996 to 1999	Prevalent HD patients	Calcitriol or paricalcitol	No treatment	NA	Historical cohort multicenter study	Time-dependent Cox model; marginal structural model	24	yes	6
Melamed et al.	2006	United States	1007	1995 to 1998	Incident HD and PD patients	Calcitriol	No treatment	NA	Prospective cohort multicenter study	Baseline and time-dependent Cox models	48	no	5
Kalantar-Zadeh et al.	2006	United States	58058	2001 to 2003	Prevalent HD patients	Paricalcitol	No treatment	NA	Historical cohort multicenter study	Baseline and time-dependent Cox models	24	no	7
Tentori et al.	2006	United States	14967	1999 to 2004	Prevalent HD patients	Calcitriol; paricalcitol; doxercalciferol	No treatment; each other	NA	Historical cohort multicenter study	Baseline and time-dependent Cox models; as treated analysis	60	yes	7
Kovesdy et al.	2008	United States	520	1990 to 2007	CKD stage 2 to 4 patients	Calcitriol	No treatment	1.75-3.5ug/week	Prospective cohort single center study	Baseline Cox model	48	no	6
Naves-Diaz et al.	2008	Argentina; Brazil; Colombia; Chile; Mexico; Venezuela	16004	2000 to 2004	Prevalent HD patients	Calcitriol or alfacalcidol	No treatment	NA	Historical cohort multicenter study	Time-dependent Cox model	54	yes	6
Shinaberger et al.	2008	United States	34307	2001 to 2004	Prevalent HD patients	Paricalcitol	No treatment	1.7-30.8ug/week	Historical cohort multicenter study	Baseline Cox model	30	no	7
Shoben et al.	2008	United States	1418	1999 to 2007	CKD stage 3 to 4 patients	Calcitriol	No treatment		Historical cohort multicenter study	Baseline Cox model; as-treated analysis	48	yes	8
Wolf et al.	2008	United States	9303	2004 to 2005	Incident HD patients	Calcitriol; paricalcitol; doxercalciferol	No treatment; stratified by race	NA	Prospective cohort multicenter study	Baseline Cox model	12	no	5
Tentori et al.	2009	France; Germany; Italy; Japan; Spain; United Kingdom; United States; Australia; Belgium; Canada; New Zealand; Sweden	38066	1996 to 2009	Incident HD patients	Calcitriol; paricalcitol; doxercalciferol	No treatment; each other	NA	Prospective cohort multicenter study	Baseline and time-dependent Cox models; Marginal structural model	30	no	5
Peter et al.	2009	United States	193830	1999 to 2004	Prevalent and incident HD	Calcitriol; paricalcitol; doxercalciferol	No treatment	0.25-3.5ug/week*	Historical cohort multicenter study	Time-dependent Cox model	63	no	6
Chang et al.	2009	Taiwan	702	1993 to 2004	Incident HD	Calcitriol	No treatment	0.75-6.0ug/week	Retrospective cohort single center study	Baseline Cox model	140	no	6
Konta et al.	2010	Japan	466	2003 to 2008	Incident HD	Calcitriol; falecalcitriol; maxacalcitol	No treatment	1.1-5.1ug/week; 1.4-2.6ug/week; 2.6-5.4ug/week	Prospective cohort multicenter study	Baseline Cox model	60	no	7
Sugiura et al.	2010	Japan	665	1992 to 2008	Incident HD	Alfacalcidol	No treatment	1.75-3.5ug/week	Retrospective cohort single center study	Baseline Cox model	132	no	6
Jean et al.	2011	France	648	2005 to 2009	Prevalent HD patients	Alfacalcidol	No treatment	1.75-9ug/week	Prospective cohort multicenter study	Baseline Cox model	42	no	5
Brancaccio et al.	2011	Italy	2378	2006 to 2007	Incident HD patients	Calcitriol; paricalcitol	No treatment	1.9-3.3ug/week; 11.2-15.9ug/week	Prospective cohort multicenter study	Time-dependent Cox model	18	no	5
Dierkes et al.	2011	Germany	650	NA	NA	Calcitrol; cholecalciferol	No treatment	NA	Prospective cohort multicenter study	NA	24	NA	NA
Ogawa et al.	2012	Japan	190	2005 to 2010	Prevalent HD patients	Alfacalcidol	No treatment	3.4-7.0ug/week	Prospective cohort single center study	Baseline Cox model	60	no	9

Asterisk (*) indicate the calcitriol equivalent doses. Paricalcitol and doxercalciferol doses were converted to calcitriol equivalent doses with ratios 4.6:1 for paricalcitol:calcitriol and 3.1:1 for doxercalciferol:calcitriol.

### Vitamin D and all-cause mortality

**Figure 2:**
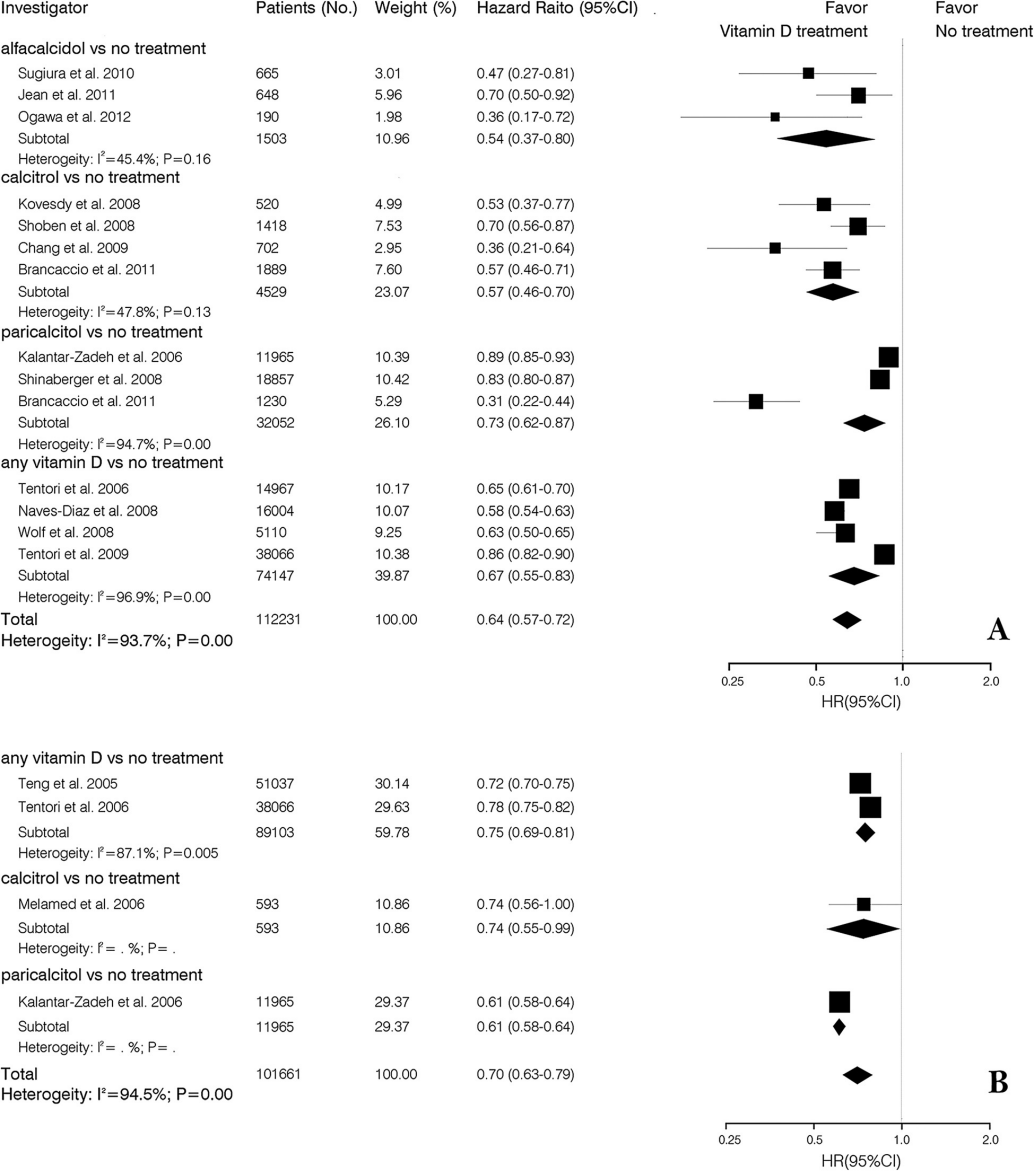
**Pooled crude hazard ratio of all-cause mortality for vitamin D treatment vs. no treatment in CKD patients. (A)** baseline Cox model; **(B)** time-dependent Cox model.

14 studies examined the association between active vitamin D treatment and crude all-cause mortality. Patients that received alfacalcidol had a 46% (HR, 0.54; 95% CI, 0.37-0.80) lower overall mortality risk compared to untreated patients. Calcitriol, paricalcitol and not otherwise specified active vitamin D treated patients had a 43% (HR, 0.57; 95% CI, 0.46-0.70), 27% (HR, 0.73; 95% CI, 0.62-0.87) and 36% (HR, 0.64; 95% CI, 0.57-0.72) lower overall mortality risk. Similar results were observed with the crude time-dependent Cox model. All-cause mortality risk with calcitriol, paricalcitol and not otherwise specified active vitamin D was 26% (HR, 0.74; 95% CI, 0.55-0.99), 39% (HR, 0.61; 95% CI, 0.58-0.64) and 30% (HR, 70; 95% CI, 0.63-0.79) lower, respectively, than that found patients without active vitamin D treatment (Figure 2).

**Figure 3:**
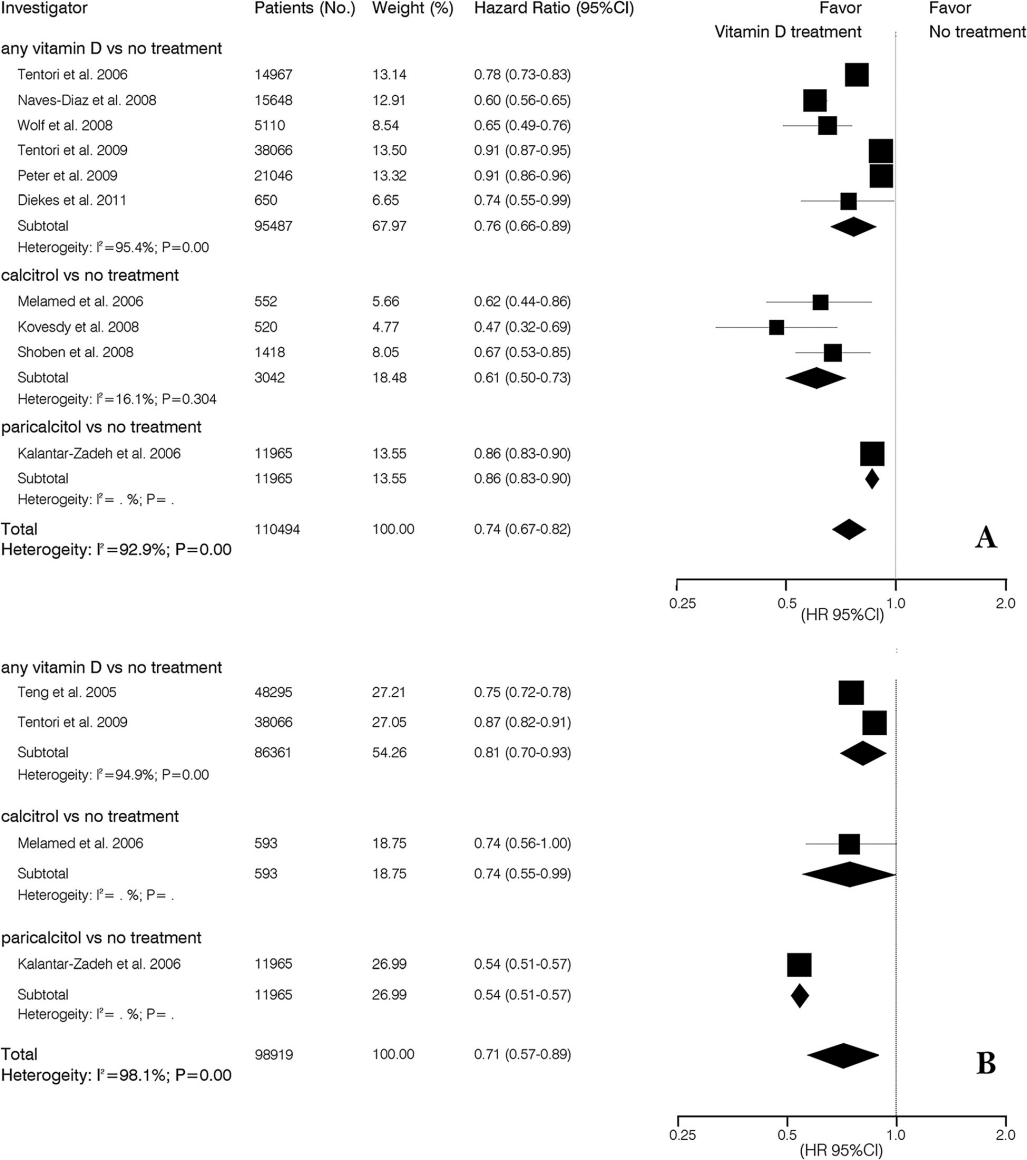
**Pooled case mixed adjusted hazard ratio of all-cause mortality for vitamin D treatment vs. no treatment in CKD patients. (A)** baseline Cox model; **(B)** time-dependent Cox model.

Ten studies reported vitamin D intake and risk for all-cause mortality using an adjusted case mixed baseline model. The risk of all-cause mortality was reduced 39% (HR, 0.61; 95% CI, 0.50-0.73) with calcitriol and 14% (HR, 0.86; 95% CI, 0.83-0.90) with paricalcitol. Using the adjusted case mixed time-dependent Cox model, patients who received active vitamin D treatment had a survival benefit (HR, 0.71; 95% CI, 0.57-0.89) (Figure 3).

We pooled data for ESRD on dialysis patients and CKD not on dialysis patients. Three studies evaluated patients with CKD that were not on dialysis. The survival advantage was similar in both the crude model (HR, 0.61; 95% CI, 0.43-0.77) and the adjusted model (HR, 0.59; 95% CI, 0.35-0.99). Patients with ESRD on dialysis had less survival benefit in the adjusted model (HR, 0.80; 95% CI, 0.63-0.94) than in the crude model (HR, 0.65; 95% CI, 0.58-0.73) (Table 2).

**Table 2:** Pooled hazard ratio for ESRD on dialysis and CKD not on dialysis

**Patient group**	**# patients**	**Hazard ratio**	**# studies**	**I**^**2**^**, %**
Patients with CKD not on dialysis				
Crude all-cause mortality	2603	0.61 (0.48–0.77)	3	29.2
Adjusted all-cause mortality	2603	0.59 (0.35–0.99)	3	79
Patients with ESRD on dialysis				
Crude all-cause mortality	109628	0.65 (0.58–0.73)	11	95
Adjusted all-cause mortality	66639	0.80 (0.68–0.94)	6	94.4

### Vitamin D and cardiovascular mortality

Four studies reported the HR between active vitamin D treatment and cardiovascular mortality using a crude Cox model and five using an adjusted baseline Cox model. A significant survival advantage was found in patients receiving active vitamin D using an unadjusted analysis (HR, 0.41; 95% CI, 0.28-0.59) and an adjusted analysis (HR, 0.59; 95% CI, 0.41-0.86). Similar results were found with calcitriol and paricalcitol. The adjusted baseline Cox model analysis found the reduction of cardiovascular mortality with calcitriol and paricalcitol to be 37% (HR, 0.63; 95% CI, 0.50-0.79) and 57% (HR, 0.43; 95% CI, 0.29-0.63), respectively. There was no survival difference associated with alfacalcidol treatment (HR, 0.45; 95% CI, 0.14-1.47) (Table 3).

**Table 3:** Pooled hazard ratio for cardiovascular mortality in patients receiving vitamin D or no treatment

**Patient groups**	**# patients**	**Hazard ratio**	**# studies**	**I2, %**
Crude cardiovascular mortality using baseline Cox model				
Alfacalcitol vs no treatment	432	0.37 (0.25–0.55)	2	0
Calcitrol vs no treatment	1889	0.57 (0.46–0.71)	1	NA
Paricalcitol vs no treatment	1230	0.31 (0.22–0.44)	1	NA
Overall	3551	0.41 (0.28–0.59)	4	69.9
Adjusted cardiovascular mortality using baseline Cox model				
Any vitamin D vs no treatment	466	0.59 (0.19–1.82)	2	68.6
Alfacalcitol vs no treatment	665	0.45 (0.14–1.47)	1	NA
Calcitrol vs no treatment	1889	0.63 (0.50–0.79)	1	NA
Paricalcitol vs no treatment	1230	0.43 (0.29–0.63)	1	NA
Overall	4250	0.59 (0.41–0.86)	5	83.6

### Calcitriol vs paricalcitol and all-cause mortality

Three studies reported hazard ratios that compared calcitriol and paricalcitol treatment. The crude baseline Cox model found a survival advantage with paricalcitol treatment (HR, 0.80; 95% CI, 0.75-0.86). In contrast, the adjusted baseline Cox case mixed and malnutrition-inflammation-cachexia syndrome (MICS) model demonstrated a survival advantage with calcitriol treatment (HR, 0.95; 95% CI, 0.91-0.99) in (Table 4).

**Table 4:** Comparison of all-cause mortality with paricaltitol and calcitrol

**Patient group**	# **patients**	**Hazard ratio**	**# studies**	**I2, %**
Crude baseline Cox model	75130	0.80 (0.75–0.86)	2	0
Adjusted baseline Cox case mixed model	16008	0.89 (0.79–1.00)	3	62.9
Adjusted baseline Cox case mixed and MICS model	14384	0.95 (0.91–0.99)	2	0

### Vitamin D dosage and all-cause mortality

Three studies reported the relationship between active vitamin D dose and all-cause mortality. Calcitriol treatment was associated with a dose dependent decrease in all-cause mortality. There was no survival advantage when calcitriol dose exceeded 7 ug per week. A dose dependent response was not found with paricalcitol (Table 5).

**Table 5:** Vitamin D dosage and all-cause mortality risk

**Investigator**	**# patients**	**Follow up (months)**	**Dosage (ug/week)**	**Mean dosage (ug/week)**	**Hazard ratio**	**95% CI**
Calcitrol						
Naves-Diaz et al.	1304	54	<1.75	1.05	0.46	0.37–0.53
Naves-Diaz et al.	1053	54	1.75-3.5	2.38	0.58	0.49–0.70
Naves-Diaz et al.	432	54	3.5-7.0	4.69	0.64	0.50–0.83
Naves-Diaz et al.	184	54	>7.0	11.83	0.83	0.58–1.19
Paricalcitol						
Kalantar-Zadeh et al.	5288	24	1.0-5.0	NA	0.53	0.50–0.57
Kalantar-Zadeh et al.	11965	24	5.0-10.0	NA	0.54	0.51–0.57
Kalantar-Zadeh et al.	8326	24	10.0-15.0	NA	0.54	0.51–0.57
Kalantar-Zadeh et al.	11816	24	>15.0	NA	0.73	0.69–0.77
Shinaberger et al.	9575	30	1.7-20.1	10.9	0.93	0.89–0.97
Shinaberger et al.	8277	30	4.6-25.8	15.2	0.88	0.84–0.94
Shinaberger et al.	5875	30	6.4-30.8	18.6	0.88	0.84–0.93

### Assessment bias and meta-regression analysis

**Figure 4:**
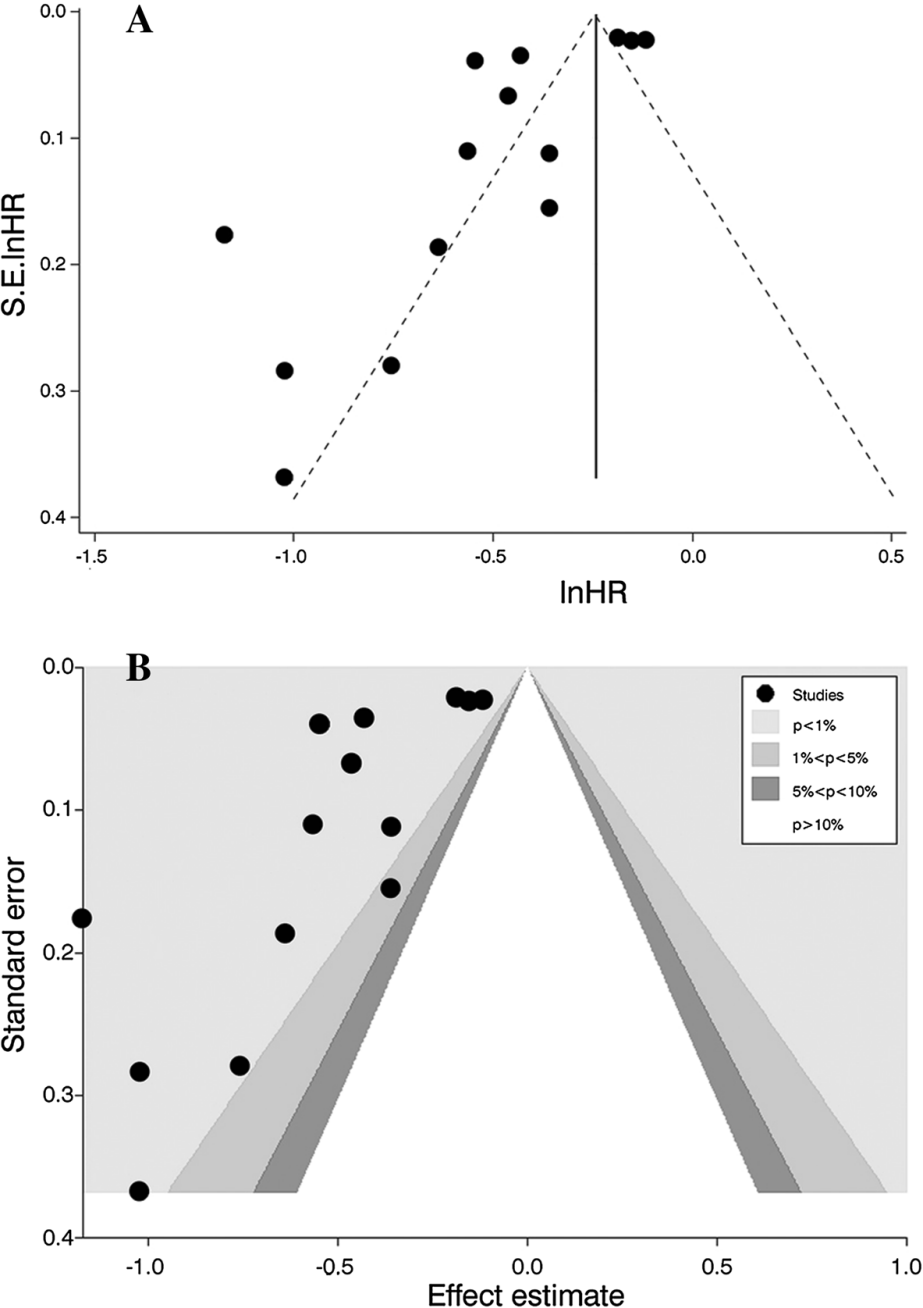
**Funnel plot and contour-enhanced funnel plot used to explore the source of publication bias. (A)** funnel plot; **(B)** contour-enhanced funnel plot.

A publication bias was identified using an Egger regression asymmetry test (β=−3.81, P=0.01) and a funnel plot (Figure 4). A contour-enhanced funnel plot was used to explore the source of the bias. The contour-enhanced funnel plot demonstrated that the majority of studies had a high statistical significance. Therefore, publication bias was a less likely cause of the funnel plot asymmetry (Figure 4).

**Figure 5:**
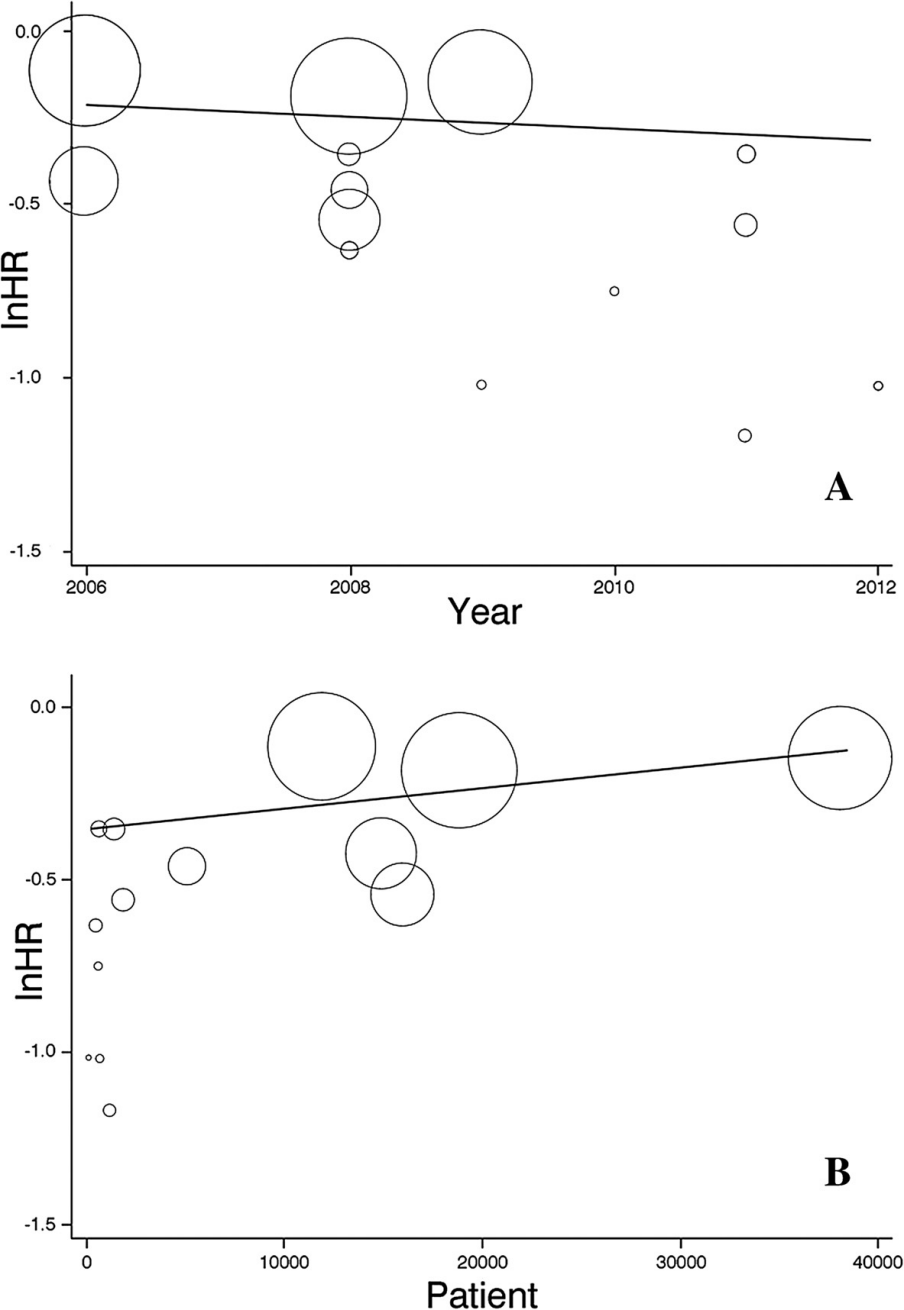
**Meta-regression graph of hazard ratio for all-cause mortality in vitamin D treated vs. no treatment patients. (A)** meta-regression by publication year; **(B)** meta-regression by number of study patients.

Within study heterogeneity was evaluated using covariate meta regression analysis. Of the seven covariates, publication year (t=−2.19, P=0.049) and study participants (t=2.52, P=0.027) had the greatest between study variance. The proportion of within-study variance explained by publication year and study participants was 24.14% and 36.20%, respectively (Figure 5).

## Discussion

Active vitamin D compounds were associated with a reduced risk of mortality in patients with ESRD on dialysis and patients with CKD not requiring dialysis. Several mechanisms may explain how vitamin D can modify risk for mortality. Vitamin D down regulates the renin-angiotensin system [45], improves insulin secretion and sensitivity [46], inhibits vascular smooth-muscle cell proliferation [47], protects normal endothelial cell function [48], modulates inflammatory processes [49], inhibits anticoagulant activity [50], and inhibits myocardial cell hypertrophy and proliferation [51]. These findings suggest that vitamin D may decrease mortality through multiple pathways. Although the actual mechanism of mortality is unclear, patient death has been associated with vascular calcifications, left-ventricular hypertrophy and left-ventricular dysfunction. The multi-organ protective effects of vitamin D may explain the lower mortality rate found in these patients.

A fixed covariate baseline Cox model was used in the majority of included studies. Only 5 studies used a time-dependent Cox model to analyze the relationship between active vitamin D use and survival. Although a standard baseline Cox proportional regression model is usually used to analyze cohort studies, it may be inadequate to evaluate active vitamin D treatments due to the presence of time-dependent variation in outcome. Higher serum calcium and phosphorus levels were consistently associated with increased risk of death [4,52]. Elevated serum PTH levels have also been associated with increased mortality [4,33]. The serum levels of calcium, phosphorus and PTH are affected by vitamin D therapy. Serum levels of calcium, phosphorus and PTH vary during the course of vitamin D therapy and affect patient outcome. These mineral metabolism indexes are recognized as time-dependent confounders. Time-dependent confounders cannot be controlled by conventional survival analysis methods [53]. Marginal structural modeling (MSM) can control for time-dependent confounders affected by prior treatment [54]. Under some conditions, the treatment estimate from a MSM can have the same causal interpretation as an estimate from a randomized clinical trial [55]. Only the Tentori et al. study reported detailed data regarding the survival advantage of patients treated with active vitamin D. The unadjusted baseline Cox model and time-varying MSM models demonstrated a 16% and 22%, respectively, reduction of all-cause mortality associated with active vitamin D treatment. Most studies included in this meta-analysis had some selection bias. For example, the study of Teng et al. [27] had statistical differences in the baseline characteristics of patient age, primary cause of renal failure, body mass index, blood pressure, and intact parathyroid hormone and hemoglobin levels. Several studies included in the meta-analysis used sophisticated statistical techniques, such as adjustment for time-dependent confounders, propensity score-matching or marginal structural models, to mimic the design of randomized controlled trials. Only the characteristics of patients that were treated with vitamin D analogues were known to the researchers. Any confounding factors would be controlled by these statistical methods and the results would be comparable to randomized controlled trials. The problem with the observational studies was that such knowledge was not available. The potential presence of unmeasured confounders prevented any conclusions of causation, even when sophisticated statistical methods were used. The survival advantage associated with active vitamin D treatment occurred in a dose-dependent manner. This phenomenon has been supported by two studies [29,32]. There has been no well-designed dose gradient study to test this hypothesis. Although we do not have higher quality evidence to prove this association, we believe that vitamin D will improve survival.

The meta-analysis detected slight differences in survival associated with different analogues of active vitamin D. The baseline case mixed and MICS Cox models detected a 5% lower mortality with paricalcitol treatment than with calcitriol treatment. This slight survival difference may be explained by differential effects of calcitriol and its analogue, paricalcitol on vascular calcification. *In vitro* studies have demonstrated that calcitriol is a growth factor for vascular smooth muscle cells, while the analogue, paricalcitol, is not [56]. *In vivo* studies have shown that vitamin D sterols have a differential effect on vascular calcification. 1-α-hydroxy vitamin D (calcitriol) was associated with greater vascular calcification than paricalcitol, even though there was equivalent suppression of PTH in these animal models [57]. Only two well-designed cohort studies or randomized controlled trials, Teng et al. [25] and Tentori et al. [30], have evaluated the mortality risk associated with different active vitamin D analogues. Further studies are needed to clarify the survival difference before one vitamin D analogue is recommended over another in clinical practice.

Three studies included in the meta-analysis reported mortality risk associated with different mean daily or weekly doses of vitamin D. In the Naves-Diaz et al. study, the maximum reduction of mortality occurred when the mean daily dose of oral calcitriol was less than 0.25 ug. This survival benefit was lost as the mean daily calcitriol dose was increased to more than 1.0 ug. This dose-dependent benefit effect was also reported with paricalcitol. Kalantar-Zadeh et al. reported patients treated with mean weekly doses of 1.0 ug to 5.0 ug of paricalcitol. Mean weekly doses of paricalcitol above 15.0 ug were associated with an 18% reduction of mortality risk. A possible explanation is that low-dose vitamin D exerts weaker anti-vascular calcification effects than higher doses in CKD patients. High doses of vitamin D could be associated with adverse effects, such as hypercalcemia, that would overwhelm its protective effects.

There were several limitations in our meta-analysis. First, only a few of the included studies used a time-dependent or marginal structural model to analyze the follow-up data. The majority of studies had limited power to draw a definitive conclusion on the effects of vitamin D supplements on all-cause or cardiovascular mortality. Second, there was high heterogeneity in the meta-analysis. Sample size and publication year were the sources of heterogeneity. Third, the possible sources of heterogeneity could not be carefully examined. This included observational studies of the use of recombinant erythropoietin to correct anemia and studies of phosphorus binders to ameliorate hyperphosphatemia in patients with CKD that showed beneficial effects on mortality, CVD outcome, and progression of renal disease. Fourth, we did not seek to identify unpublished studies and several studies were excluded because the published data were not suitable for meta-analysis.

## Conclusions

Active vitamin D compounds used to treat abnormal calcium, phosphorus and PTH levels in patients with either ESRD on dialysis or CKD not requiring dialysis. Active vitamin D compound treatment was associated with decreased all cause and cardiovascular mortality. Low dose active vitamin D compounds were associated with improved survival. Large, well designed randomized trials of active vitamin D supplements with different doses are needed to elucidate the role of vitamin D supplementation in reducing mortality.

## Competing interests

The authors declare that they have no competing interests.

## Authors’ contributions

ZZ and HS are responsible for the literature assessment and data analysis. JJ and DL contributed to the literature search and manuscript drafts. SL was a designer of entire study. All authors read and approved the final manuscript.

## Pre-publication history

The pre-publication history for this paper can be accessed here:

http://www.biomedcentral.com/1471-2369/14/199/prepub
